# A novel framework for fully automated co-registration of intravascular ultrasound and optical coherence tomography imaging data

**DOI:** 10.1093/ehjdh/ztag007

**Published:** 2026-01-16

**Authors:** Xingwei He, Kit Mills Bransby, Ahmet Emir Ulutas, Thamil Kumaran, Nathan Angelo Lecaros Yap, Gonul Zeren, Hesong Zeng, Yao-Jun Zhang, Ryota Kakizaki, Yasushi Ueki, Jonas Häner, George C M Siontis, Sylvain Losdat, Andreas Baumbach, James Moon, Anthony Mathur, Ryo Torii, Jouke Dijkstra, Qianni Zhang, Lorenz Räber, Christos V Bourantas

**Affiliations:** Department of Cardiology, Barts Heart Centre, Barts Health NHS Trust, London, UK; Division of Cardiology, Department of Internal Medicine, Tongji Hospital, Tongji Medical College, Huazhong University of Science and Technology, Wuhan, China; School of Electronic Engineering and Computer Science, Queen Mary, University of London, London, UK; Centre for Cardiovascular Medicine and Devices, William Harvey Research Institute, Queen Mary University London, London, UK; Centre for Cardiovascular Medicine and Devices, William Harvey Research Institute, Queen Mary University London, London, UK; Department of Cardiology, Barts Heart Centre, Barts Health NHS Trust, London, UK; Centre for Cardiovascular Medicine and Devices, William Harvey Research Institute, Queen Mary University London, London, UK; Division of Cardiology, Department of Internal Medicine, Tongji Hospital, Tongji Medical College, Huazhong University of Science and Technology, Wuhan, China; Department of Cardiology, Xuzhou Third People’s Hospital, Xuzhou, China; Department of Cardiology, Bern University Hospital, University of Bern, Bern, Switzerland; Department of Cardiology, Bern University Hospital, University of Bern, Bern, Switzerland; Department of Cardiology, Bern University Hospital, University of Bern, Bern, Switzerland; Department of Cardiology, Bern University Hospital, University of Bern, Bern, Switzerland; CTU Bern, University of Bern, Bern, Switzerland; Department of Cardiology, Barts Heart Centre, Barts Health NHS Trust, London, UK; Centre for Cardiovascular Medicine and Devices, William Harvey Research Institute, Queen Mary University London, London, UK; Department of Cardiology, Barts Heart Centre, Barts Health NHS Trust, London, UK; Institute of Cardiovascular Sciences, University College London, London, UK; Department of Cardiology, Barts Heart Centre, Barts Health NHS Trust, London, UK; Centre for Cardiovascular Medicine and Devices, William Harvey Research Institute, Queen Mary University London, London, UK; Department of Mechanical Engineering, University College London, London, UK; Division of Image Processing, Department of Radiology, Leiden University Medical Center, Leiden, The Netherlands; School of Electronic Engineering and Computer Science, Queen Mary, University of London, London, UK; Department of Cardiology, Bern University Hospital, University of Bern, Bern, Switzerland; Department of Cardiology, Barts Heart Centre, Barts Health NHS Trust, London, UK; Centre for Cardiovascular Medicine and Devices, William Harvey Research Institute, Queen Mary University London, London, UK

**Keywords:** Deep learning, Intravascular imaging, Co-registration, Coronary artery disease

## Abstract

**Aims:**

To develop a deep-learning (DL) framework that enables fully automated longitudinal and circumferential co-registration of intravascular ultrasound (IVUS) and optical coherence tomography (OCT) images.

**Methods and results:**

Data from 230 patients (714 vessels) with acute myocardial infarction that underwent near-infrared spectroscopy IVUS and OCT imaging in their non-infarct related vessels were analysed. Experts annotated the lumen borders (61 655 IVUS and 62 334 OCT frames), the side branches and the calcific tissue (10 000 IVUS and 10 000 OCT frames each). This information was used to train DL models that extracted these features that were then used by a dynamic time warping algorithm to co-registered longitudinally the IVUS and OCT images. The circumferential registration of IVUS and OCT was performed through a rotation cost matrix and dynamic programming. On a test set of 22 patients (77 vessels), the DL method showed high concordance with the expert analysts for the longitudinal and circumferential co-registration of the two datasets (concordance correlation coefficient >0.99 and >0.90, respectively). The Williams Index was 0.96 for longitudinal and 0.97 for circumferential alignment, indicating a comparable performance of the proposed framework to the analysts. The time needed for the DL pipeline to process imaging data from a vessel was <90 s.

**Conclusion:**

A fully automated, DL-based framework for IVUS–OCT co-registration demonstrated both speed and accuracy, with performance comparable to that of expert analysts. These features enable its application in research using large-scale data incorporating multimodality imaging.

## Graphical Abstract

**Graphical Abstract:**
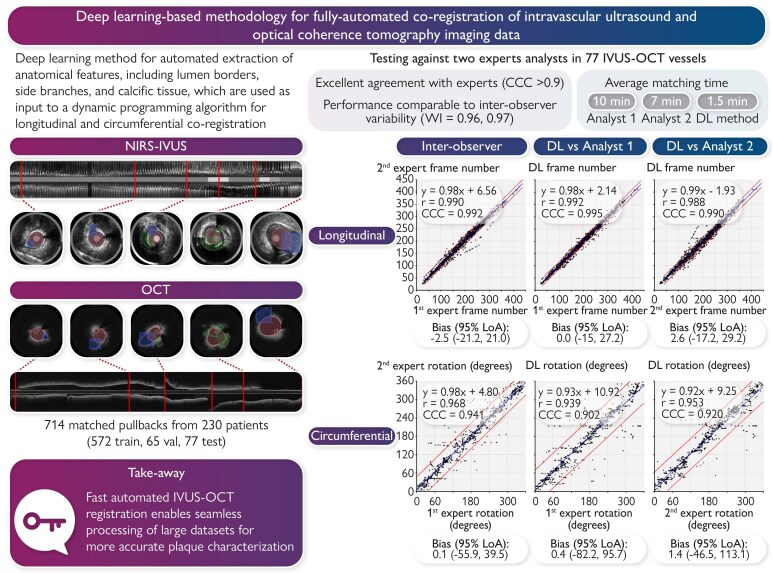

## Introduction

Intravascular imaging was introduced to assess plaque pathology *in vivo* and identify lesions that are likely to progress and cause events.^1,2^ Prospective studies using intravascular ultrasound (IVUS), optical coherence tomography (OCT) or near-infrared spectroscopy (NIRS)-IVUS have shown that the existing invasive imaging techniques are able to detect high-risk plaques with, however, a moderate positive predictive value fact that has been attributed to the inherited limitations of these modalities to assess plaque characteristics.^3–7^ This has also been confirmed in histology studies which underscored the weakness but also the complementary strengths of the existing imaging techniques to assess plaque morphology.^8,9^ In an attempt to overcome these limitations hybrid imaging catheters have been introduced that combine different imaging techniques in a single probe and appear superior to standalone imaging in assessing plaque composition and architecture; however, the clinical applications of these approaches are limited and today there is no imaging system that combines the three clinically used modalities (IVUS, OCT and NIRS) in a single probe.^10^

Therefore, there is a trend over the recent years to perform multimodality intravascular imaging in studies that aim to thoroughly assess plaque pathology or to examine the implications of novel pharmacotherapies on atherosclerotic disease progression and regression.^11–15^ In these studies, the collected data are analysed separately as their co-registration requires expertise and is a laborious process. To overcome these limitations, we present in this study a fully automated deep-learning (DL)-based framework for feature extraction and co-registration of IVUS and OCT images.

## Methods

### Study population

We retrospectively analyse data from 230 patients (714 vessels) collected in the PACMAN-AMI trial (NCT03067844), an investigator-initiated, multi-centre, randomized, double-blind clinical trial conducted at nine centres in four European countries that aimed to investigate the effect of intensive lipid-lowering therapy with alirocumab, added to high-intensity statin therapy on plaque characteristics in patients admitted with an acute myocardial infarction (AMI).^12,16^ The recruited patients had successful percutaneous coronary intervention (PCI) of the culprit lesion and angiographic evidence of coronary artery disease without a significant obstruction (diameter stenosis >20% and <50% by visual estimate) in the proximal segment of the non-culprit vessels and high low density lipoprotein measured before intervention (≥125 mg/dL for patients that were not receiving a stable statin dose for at least 4 weeks or ≥70 mg/dL for those that were on a stable statin dose for at least 4 weeks). All patients underwent NIRS-IVUS and OCT imaging in the non-culprit vessels post PCI and were then randomized at 1:1 ratio to high-intensity statin therapy and subcutaneous alirocumab therapy (150 mg biweekly) or statin monotherapy plus placebo. Treatment was given for 52 weeks and then the patients were invited for repeat angiography, NIRS-IVUS and OCT imaging of the non-culprit vessels. The study protocol complied with the Declaration of Helsinki and was approved by the local research ethics committee. All patients provided informed consent for enrolment in the institutional database for potential future investigations.

### NIRS-IVUS and OCT images data acquisition

NIRS-IVUS and OCT imaging were performed in the proximal segments of the non-culprit vessels for a length of at least 50 mm using the 40 MHz INSIGHT TVC-C195-22 or the 50 MHz INSIGHT TVC.C195-32 catheter (Infraredx, a Nipro Company, Burlington, MA, USA). After intracoronary administration of 100–200μg of nitroglycerine the catheter was advanced to the distal vessel and then was pulled back by an automated pull-back device at a speed of 0.5 mm/s. In each patient, the same system was used to assess the non-culprit vessels at baseline and follow-up.

OCT imaging was performed in the segments assessed by NIRS-IVUS using the ILUMIEN OPTIS (Abbott Vascular Santa Clara, CA, USA) system. After administration of intracoronary nitroglycerin, the OCT catheter was advanced to the distal vessel and pulled-back during contrast injection at a speed of 36 mm/s, acquiring 180 frames per second. For optimal image quality contrast injection was performed using an automated ACIST CVi Contrast Delivery System with an injection rate of ≥5.0 mL/s for the left coronary system and ≥4.0 mL/s for the right coronary artery depending on the vessel size.

### NIRS-IVUS and OCT segment extraction

NIRS-IVUS and OCT analysis was performed at the Coronary Imaging Corelab Queen Mary University London. An expert analyst (C.V.B.) reviewed the intravascular imaging data acquired at baseline and follow-up and used anatomical landmarks such as the coronary ostia or the origin of side branchers seen in the angiographic datasets, the NIRS-IVUS and the OCT images to define the segment of interest (SOI) that consisted of the longest segment that was assessed by both NIRS-IVUS and OCT at the two time points. NIRS-IVUS and OCT data with poor image quality that did not allow visualization of the vessel wall were excluded from the analysis. The NIRS-IVUS images portraying the SOI were then processed using a dedicated deep-learning algorithm,^17^ that enables retrospective identification of the end-diastolic (ED) frames, while the OCT frames were analysed in the SOI at every 0.4 mm interval. The ED frame detection in the NIRS-IVUS is an important step for the accurate co-registration of the two datasets as the NIRS-IVUS probe—that was pulled-back at a slow speed—moves back and forward in relation to the vessel during the cardiac cycle; as a consequence, it is likely consecutive NIRS-IVUS frames do not portray consecutive segments of the vessel. The analysis of the ED frames in NIRS-IVUS overcomes this limitation and is expected to facilitate the co-registration of the IVUS and OCT data where the back-forward motion of the catheter is less prominent as the OCT catheter is pulled-back at a faster speed.^18^ The 714 SOI identified in 230 patients were then split in 3 sets: a feature training set consisted of 187 patients (572 vessels), a validation set consisted of 21 patients (65 vessels), and a test set consisted of 22 patients (77 vessels).

### IVUS and OCT manual co-registration

**Figure 1:**
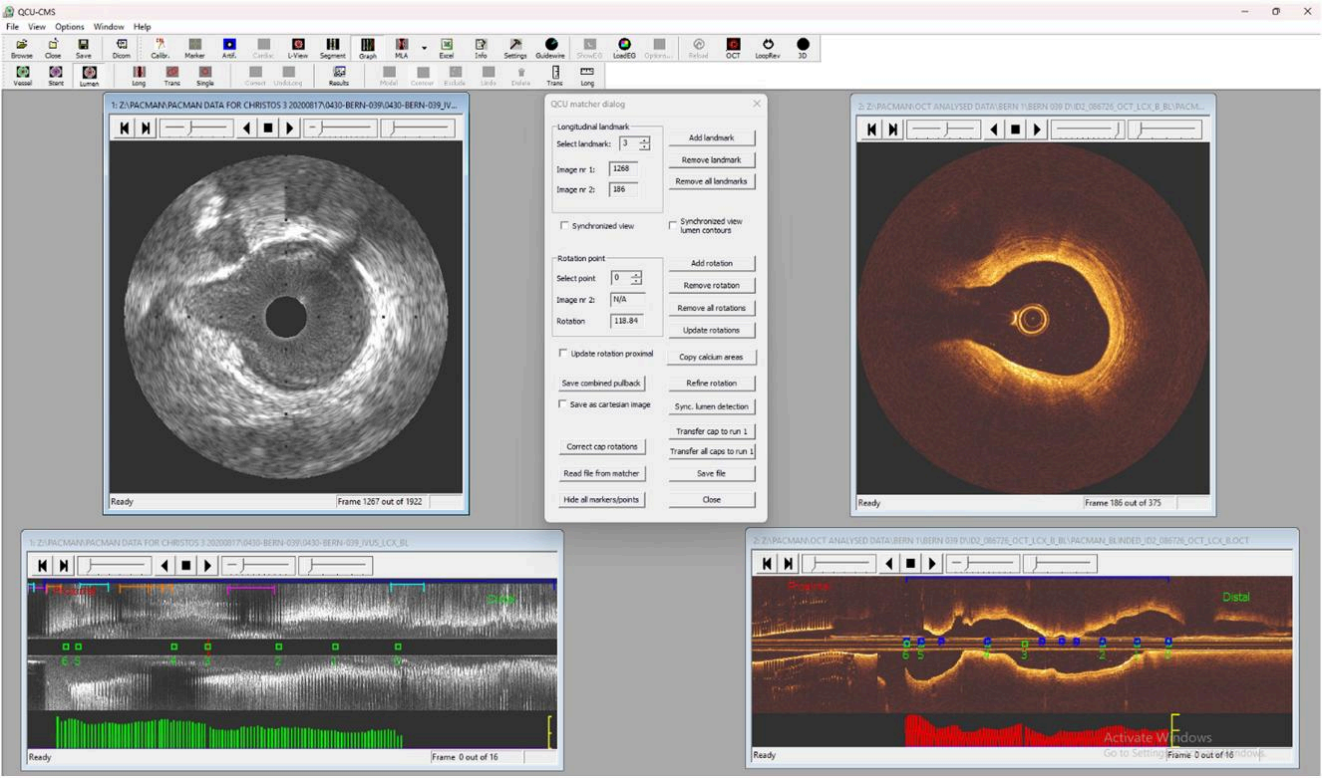
Snapshot of QCU-CMS software used by the expert to co-register the NIRS-IVUS and OCT data. After the identification of the ED frames in NIRS-IVUS, showing in the longitudinal IVUS image with green bars, and the selection of OCT frames at 0.4 mm interval, shown in the longitudinal OCT image with red bars, in the SOI an expert analyst reviewed the IVUS and OCT images and identify corresponding frames using anatomical landmarks (side branches, and the presence of calcification). The location of these landmarks is indicated with a green dot and a number in the longitudinal IVUS and OCT frame. These landmarks were also used to define the circumferential co-registration in the NIRS-IVUS and OCT images. The frames where the expert rotated the OCT images to circumferentially align them with IVUS are shown in the longitudinal OCT image with a blue dot. ED, end diastolic; SOI, segment of interest.

Data consisting the validation (21 patients, 65 vessels) and test sets (22 patients, 77 vessels) were selected for manual co-registration by expert analysts. First, an expert analyst (A.U.) reviewed the NIRS-IVUS and OCT images consisting of the SOI in these sets, identified corresponding frames and estimated their rotational orientation. A specially designed module of the QCU-CMS software (Version 4.69, Leiden University Medical Center, Leiden, the Netherlands) was used for simultaneous visualization of the NIRS-IVUS and OCT pullbacks allowing the expert analyst to identify matched sections that portray anatomical landmarks such as the origin of side branches or the presence of calcific tissue that were visible in both modalities (*Figure 1*). In the test set matching was also performed by a second analyst and the first analyst performed the analysis twice so as to report the inter- and intra-observer variability. The lipid core tissue distribution was not considered in the matching process of the two modalities as there were often differences in the output of these two techniques. Linear interpolation was then applied to match the NIRS-IVUS and OCT images located between corresponding sections. In this way, each NIRS-IVUS cross section of the SOI had a corresponding OCT frame. In a final step, the anatomical landmarks that were used to match NIRS-IVUS and OCT images were also used to identify the circumferential orientation of the OCT in relation to NIRS-IVUS, and then, the OCT images were rotated to achieve optimal longitudinal and circumferential alignment of the two datasets. The matched NIRS-IVUS and OCT images were then used as ground truth to validate and test the effectiveness of the DL-solution for fully automated longitudinal and circumferential co-registration of two imaging sets.

### NIRS-IVUS and OCT manual feature annotation

In the feature training set, expert analysts (X.H., A.U., and N.L.Y.) manually delineated the lumen borders in all ED NIRS-IVUS and OCT frames depicting the SOI. A subset of 10 000 frames from each modality was randomly sampled. In these frames, all side branches that intersected the lumen—irrespective of their size—were identified and marked with a rectangular bounding box; challenging cases where the side branch was partially masked by the guidewire or by other artefacts seen in NIRS-IVUS and OCT were included for training. In addition, the presence and lateral extend of calcific tissue was detected and represented with a label that indicated its presence or absence for every circumferential angle around the lumen centroid (see Supplementary material online, *Figure S1*).

The same process was followed in the validation and test set for all SOI frames. Manual feature annotation was performed after the co-registration of the NIRS-IVUS and OCT images so that analysts were blinded to the feature labels during the co-registration process. These labels were then used to train and validate DL methods for the automated detection of these features (see Supplementary material online, *Table S1*).

### Deep-learning methodology for IVUS and OCT co-registration

**Figure 2:**
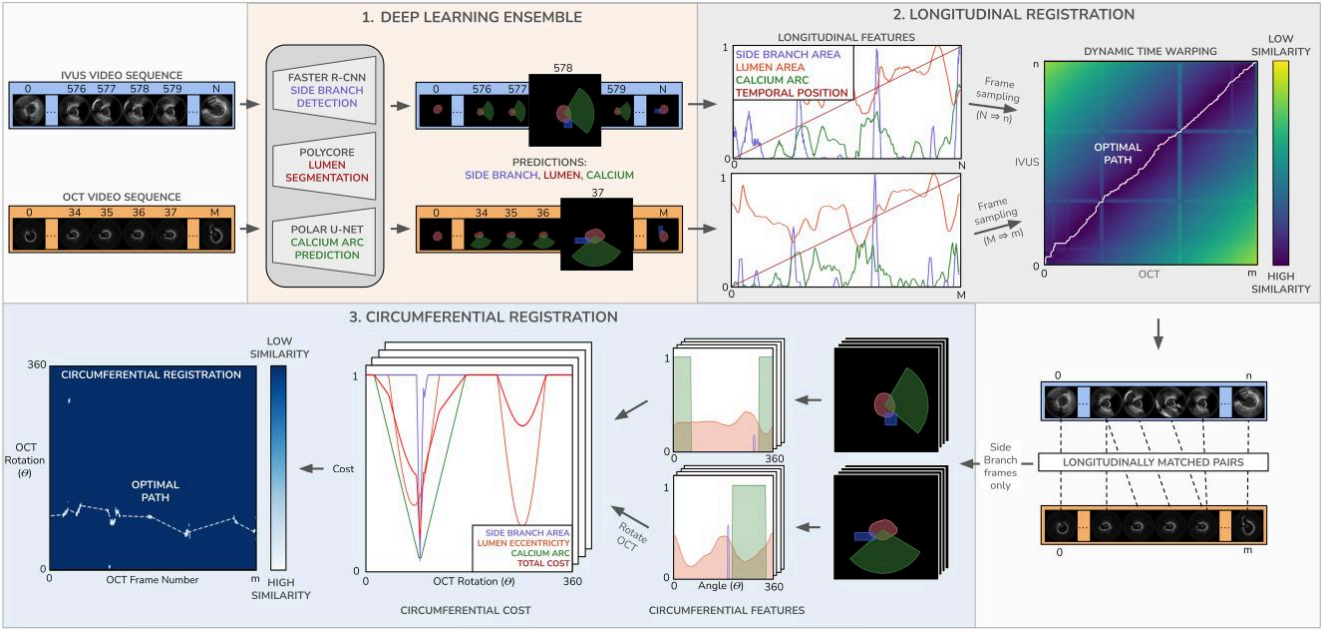
Schematic diagram of framework used to co-register the IVUS and OCT images. Features are extracted from NIRS-IVUS and OCT pullbacks using an ensemble of DL networks and these are then used for longitudinal registration using a DTW, and circumferential registration using a path finding algorithm. DL, deep learning; DTW, dynamic time warping.

In contrast to the methodologies presented in the literature, the proposed methodology was designed to process IVUS and OCT images that have not been segmented and used a multi-purpose ensemble of DL networks to extract features from the imaging data and then identify corresponding frames. A schematic diagram describing the full feature extraction and co-registration process is presented in *Figure 2*.

### Deep-learning feature extraction

The feature extraction pipeline automatically identifies the lumen borders, the origin of the side branch, and the presence of calcific tissue in IVUS and OCT using expert estimations (defined in Section 2.5) as supervision. Lumen border detection in IVUS and OCT was performed using a previously validated convolutional neural network segmentation model.^19^ This segmentation network was specifically designed to minimize the impact of image artefacts by using a topological prior, and was originally trained from data acquired with the 2.4F high-resolution (35–65 MHz) Makoto™ NIRS-IVUS Imaging System (Infraredx, a Nipro Company, Burlington, MA). Because the image data used in this study were acquired with different catheters, the model was retrained for NIRS-IVUS and OCT segmentations, using the annotations provided by expert analysts in the feature training set consisted of 61 665 NIRS-IVUS and 62 334 OCT frames. Frames portraying common artefacts seen in IVUS and OCT that did not impede the visualization of the vessel wall were included in the training set. Next, a fast methodology for automated detection of the origin of the side branches in IVUS and OCT was developed taking advantage of the expert annotations in 10 000 frames in each modality.^20^ Histology studies indicate that both IVUS and OCT have a high-diagnostic accuracy for the detection of calcific tissue; therefore, we use this information to match IVUS and OCT and develop a Polar-UNet network for the automated detection of the calcific tissue.^8^ For this network, the annotations of experts in 10 000 NIRS-IVUS frames and 10 000 OCT frames were used as input. The encoder accepts a polar view of the input image with radial and angular dimensions, while the decoder performs self-attention before classifying whether each circumferential angle around the lumen centre contains calcium using expert estimations as supervision.^21^ Schematic diagrams for all three networks in the feature extraction pipeline are presented in Supplementary material online, *Figure S2*.

To optimize the networks, cross-entropy and dice losses were combined for lumen segmentation, a smooth L1 and cross entropy losses were used for side-branch detection, and cross-entropy loss was used for the identification of the calcific arc. Each model was trained for 200 epochs using a batch size of 64, with spatial and pixel-level augmentation applied to the training input images including random rotations and random adjustments to brightness and contrast. The validation set, comprising of the matched NIRS-IVUS and OCT frames of the SOI frames from 65 vessels, was used to tune network hyperparameters and select model weights based on the epoch with the lowest loss. The description of the hyperparameters and training recipe is presented in the Supplementary material (see Supplementary material online, *Table S2*). The computer used to train the model contained a Nvidia A100 GPU with a 12-Core Intel Xeon CPU.

### Automated longitudinal matching of IVUS and OCT data

The detected lumen borders, side branches, and calcific tissue distribution in IVUS and OCT vary longitudinally, and this information was used as input to the registration modules. To not miss the geometrical information provided in frames located between ED frames we analysed all the IVUS and OCT data of the SOI, and extracted four features from each modality: (i) the lumen area; (ii) the side branch area, both areas were normalized to the [0, 1] interval using the maximum lumen area observed in the vessel; (iii) degree of calcification, quantified as the proportion of the lumen’s circumference exhibiting calcified tissue; and (iv) normalized frame position, representing the relative temporal location of each frame. To reduce noise and inter-frame variability caused by image artefacts, Gaussian smoothing was applied to all features along the longitudinal axis. This means that the feature signal from a given frame propagates to neighbouring frames; therefore, if a landmark is missed in NIRS-IVUS and OCT this will not substantially affect the matching in the SOI as co-registration will be approximated by neighbouring anatomical landmarks identified in both modalities. The sequences were subsequently down-sampled by retaining the ED frames in IVUS and every second frame in OCT. Dynamic time warping (DTW) was then performed to find the optimal longitudinal alignment between the IVUS and OCT sequence.^22^ First, a distance matrix was computed between all sampled frames in the sequences using a feature-weighted Euclidean distance. A DTW cost matrix was then defined which describes the total minimum cumulative cost of assigning the two sequences and the optimal path of IVUS-OCT frame pairs is obtained by tracing back through the matrix. The full description of the DTW algorithm and the hyperparameters tuning in the validation set is described in the Supplementary material.

### Automated circumferential registration of IVUS and OCT data

To circumferentially register paired frames automatically, we mimic the strategy of the expert analysts focusing on pairs that contain side branches, or the presence of calcific tissue and we linearly interpolated the rotations for the remaining OCT frames. In these frames we extract features that vary circumferentially such as side branch angle, calcification angle and lumen eccentricity. A circular sampling process is defined around the lumen centre, along 360° directions in 2° increments. For each direction we define the distance from lumen centroid to the lumen boundary, the side branch area, and a binary value indicating the presence of calcium in IVUS and OCT. A rotation cost matrix R is then computed by rotating every OCT frame around each angle increment and computing the feature-weighted normalized cross-correlation with its IVUS pair. For circumferential registration, we follow an established dynamic programming method^23^ where a rotational cost matrix is computed, and the lowest cost path is found by backtracking from the final to first row. The rotational movement of the path between consecutive frames is constrained by a shape regularization term developed by Zahnd *et al.*^24^. Hyperparameters tuning was performed in the validation set and is described in the Supplementary material.

### Statistical analysis

The distribution of continuous variables was assessed using the Kolmogorov–Smirnov test; a non-normal distribution was found and therefore results are presented as median (interquartile range) while categorical values are shown as absolute values and percentages. Comparisons between continuous variables were performed using the Mann–Whitney *U* test. Categorical variables were compared using the chi-square test. The performance of the feature extraction tasks was evaluated on the test set. Specifically, the performance of the lumen segmentation method was evaluated by measuring the agreement with the experts using linear regression analysis, the Spearman (*r*) correlation coefficient, and Bland–Altman analysis and by comparing the annotations of the experts and the DL method using the dice similarity coefficient (DSC) and Jaccard coefficient (JC). The global average precision (AP) metric was used to evaluate the side branch detection method while the global precision, recall and F1-score of the positive class and AP were implemented for the calcific tissue classification.^25,26^

To examine the efficacy of the developed method to accurately identify matched IVUS and OCT frames we used the estimations of the two experts in the test set as a reference standard. Specifically, for every matched OCT frame, we computed the distance in mm and the rotational orientation in degrees between the estimations of the analysts and the DL method. A Wilcoxon signed-rank test was used to determine whether there is significant difference between the differences in the estimations between experts and the differences between the experts and the proposed method. Linear regression analysis, the concordance correlation coefficient (CCC),^27^ the Spearman correlation (*r*), and the Williams Index (WI)^28^ along with its 95% confidence interval were used to evaluate the agreement between the DL method and the two experts. The WI measures whether the model’s predictions are as similar to the human experts as the experts are with each other. A WI greater than 1 suggests that the model agrees with the experts more than the experts agree with one another. Statistical analyses were performed using Python. a *P* value < 0.05 was considered a statistically significant difference.

## Results

### Studied patients

The baseline demographics of the patients included in the training, validation and test set are shown in *Table 1*. Overall, there were no significant differences between patient groups in the baseline demographics. The median number of sampled frames in the SOI were 105 (79, 134) for NIRS-IVUS and 111 (86, 133) for OCT, which corresponds to a SOI length of 44.4 (34.4, 53.2) mm.

**Table 1:** Baseline demographics of studied patients and vessels

	Studied patients (*n* = 230)	Training set (*n* = 187)	Validation set (*n* = 21)	Test set (*n* = 22)	*P*-value
Age (years)	58 (52, 64)	58 (52, 65)	54 (49, 64)	54 (50, 61)	0.652
Gender (male)	194 (84.3%)	157 (84.0%)	19 (90.5%)	18 (81.8%)	0.696
Current smoker	110 (47.8%)	91 (48.7%)	9 (42.8%)	10 (45.5%)	0.856
Family history of CAD	80 (34.8%)	64 (34.2%)	7 (33.3%)	9 (40.1%)	0.815
Co-morbidities					
Diabetes	21 (9.1%)	15 (8.0%)	4 (19.4%)	2 (9.1%)	0.251
Hypertension	97 (42.2%)	79 (42.2%)	10 (47.6%)	8 (36.3%)	0.756
Previous PCI	7 (3.0%)	5 (2.7%)	2 (9.5%)	0 (0%)	0.152
Studied vessels	714	572	65	77	0.951
LAD/diagonal branch	219 (30.7%)	178 (31.1%)	19 (29.2%)	22 (28.6%)	
LCX/intermediate/obtuse marginal	264 (37.0%)	213 (37.2%)	23 (35.4%)	28 (36.4%)	
RCA	231 (32.4%)	181 (31.6%)	23 (35.4%)	27 (35.1%)	
Matched IVUS and OCT frames	77 627	61 665	6863	9099	0.999
LAD/diagonal branch	25 789 (33.2%)	20 807 (33.7%)	2243 (32.7%)	2739 (30.1%)	
LCX/intermediate/obtuse marginal	25 333 (32.6%)	20 379 (33.0%)	2123 (30.9%)	2831 (31.1%)	
RCA	26 505 (34.1%)	20 479 (33.2%)	2497 (36.4%)	3529 (38.8%)	

CAD, coronary artery disease; LAD, left anterior descending coronary artery; LCx, left circumflex; IVUS, intravascular ultrasound; OCT, optical coherence tomography; PCI, percutaneous coronary intervention; RCA, right coronary artery.

### Efficacy of the DL methodology to detect lumen borders, side branches and calcific tissue

The estimations of the DL method were close to the estimations of the experts for the lumen areas in both NIRS-IVUS [7.38 (5.26, 10.28) mm² vs. 7.33 (5.20, 10.19) mm²; median difference: 0.16 (0.06, 0.36) mm²] and OCT [6.99 (5.09, 9.87) mm² vs. 6.99 (5.09, 9.83) mm²; median difference: 0.08 (0.04, 0.16) mm²]. The difference in the lumen areas estimated by NIRS-IVUS and OCT should be attributed to the fact that NIRS-IVUS tends to overestimate the luminal dimensions compared to OCT.^29^ A high correlation was noted between the output of the DL and the experts in both datasets (*r* = 0.991, *P* < 0.001 and *r* = 0.996, *P* < 0.001, respectively) while Bland-Altman analysis showed a small bias and narrow limits of agreement (see Supplementary material online, *Figure S3*). In addition, the DSC and JC were greater than 0.95 for the estimations of the expert and the developed DL methodology in both NIRS-IVUS and OCT (*Table 2*). In addition, the DSC and JC were greater than 0.95 for the estimations of the expert and the developed DL methodology in both NIRS-IVUS and OCT (*Table 2*). In addition, the DSC and JC were greater than 0.95 for the estimations of the expert and the developed DL methodology in both NIRS-IVUS and OCT (*Table 2*). With regards to the side branch detection, the AP was numerically higher in OCT indicating that the DL solution introduced for the detection of the side branches performed better in the OCT data. In NIRS-IVUS images, the AP was 0.58 suggesting that the DL solution had also good performance in identifying the origin of side branches in that dataset. The better performance in OCT for both lumen and side branch detection should be attributed to the fact that in OCT the lumen is more clearly visible than in NIRS-IVUS where the blood often has similar echogenic properties with the vessel wall. Finally, for the calcific tissue detection the precision, recall and F1 score were close to 0.9 in NIRS-IVUS and numerically smaller in OCT. The lower performance of the DL method to detect the calcific tissue in OCT should be attributed to the fact that this tissue type has similar optical characteristics with the lipid tissue and is difficult to be differentiated from other tissue types when this is deeply embedded.

**Table 2:** Performance metrics of the DL methods developed for the detection of the lumen borders, the side branch location, and the calcific tissue in NIRS-IVUS and OCT frames

	Lumen segmentation			Side branch detection	Calcium arc classification			
	Area error (mm^2^)	DSC	JC	AP	Precision	Recall	F1	AP
NIRS-IVUS	0.15 (0.06, 0.33)	0.98 (0.96, 0.98)	0.95 (0.93,0.97)	0.58	0.87	0.89	0.88	0.95
Oct	0.08 (0.04, 0.16)	0.98 (0.98, 0.99)	0.97 (0.96, 0.98)	0.74	0.70	0.66	0.68	0.73

DL, deep learning; NIRS-IVUS, near-infrared spectroscopy-intravascular ultrasound; OCT, optical coherence tomography; DSC, dice similarity coefficient; JC, Jaccard coefficient; AP, average precision; FI, F1-score.

### Intra- and inter-observer variability

**Figure 3:**
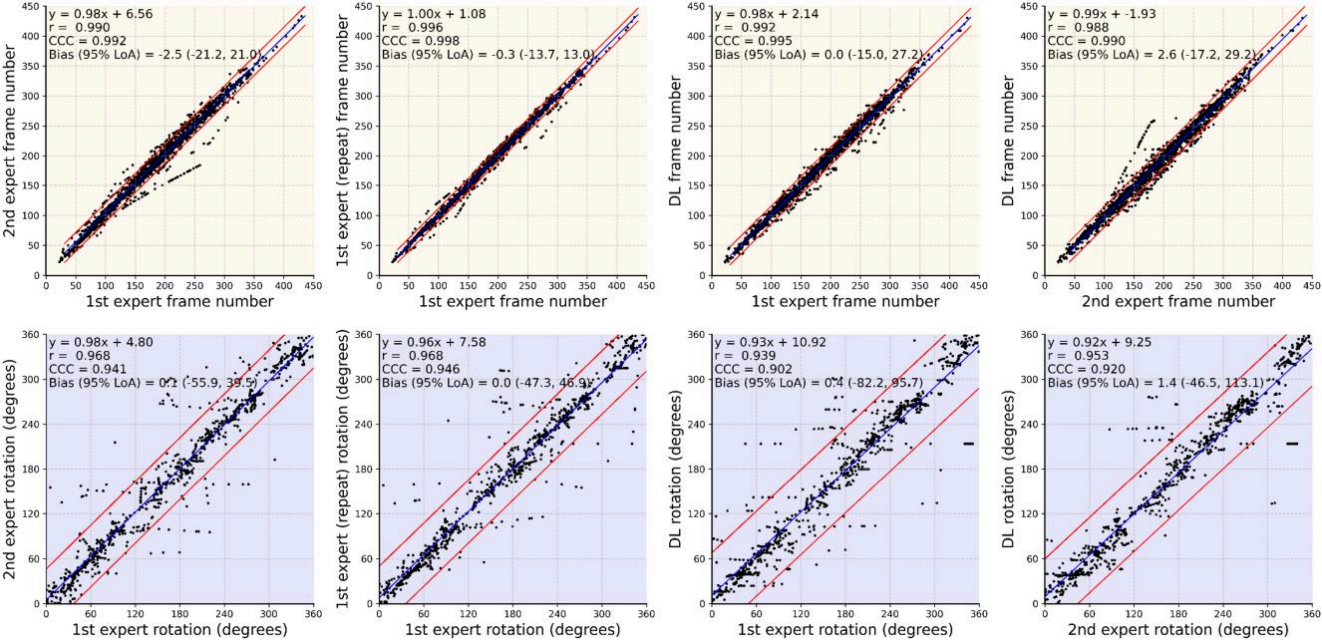
Linear regression analysis between the estimations of the experts (*A*, A’) the first and second annotation of the first expert, (*B*, B’) the estimations of the DL method and the 1st expert (C and C’) and the estimations of the DL method and the second expert (D and D’) for the longitudinal (top panels) and circumferential orientation (bottom panels) of the intravascular imaging data. The blue line represents the regression line and the black lines the limits of agreements (±1.96 SD). The Spearman correlation coefficient (*r*) and CCC values are also displayed. DL, deep learning.

The intra- and inter-observer variability of the expert analysts for the longitudinal and circumferential registration of the NIRS-IVUS and OCT frames in the test set is shown in *Table 3*. A median difference of 4.5 frames was reported between experts for the longitudinal registration, and of 9.8^o^ for the circumferential registration. The intra-observer variability for these metrics was 2.3 frames and 7.2^°^, respectively. A high CCC and Spearman correlation were noted for the expert annotations while linear regression analysis indicated a slope close to 1 and a y-intercept close to 0 for both longitudinal and circumferential registration (*Figure 3*).

**Table 3:** Longitudinal and circumferential registration results

	A1 vs. A2	A1 vs. A1*	DL vs. A1	DL vs. A2	WI (95% CI)
OCT frame difference	4.5 (2.0, 8.4)	2.3 (1.1, 3.9)	4.1 (3.2, 6.4)	5.3 (3.9, 8.6)	0.96 (0.94, 1.00)
OCT angle difference (^O^)	9.8 (6.2, 15.9)	7.2 (5.0, 12.6)	9.9 (6.7, 18.6)	10.2 (6.3, 14.8)	0.97 (0.96, 0.99)

Inter-frame spacing in OCT is 0.2 mm. DL, deep learning; OCT, optical coherence tomography; WI, Williams index; CI, confidence interval.

### Comparison of the estimations of the expert analysts and the DL-methodology

**Figure 4:**
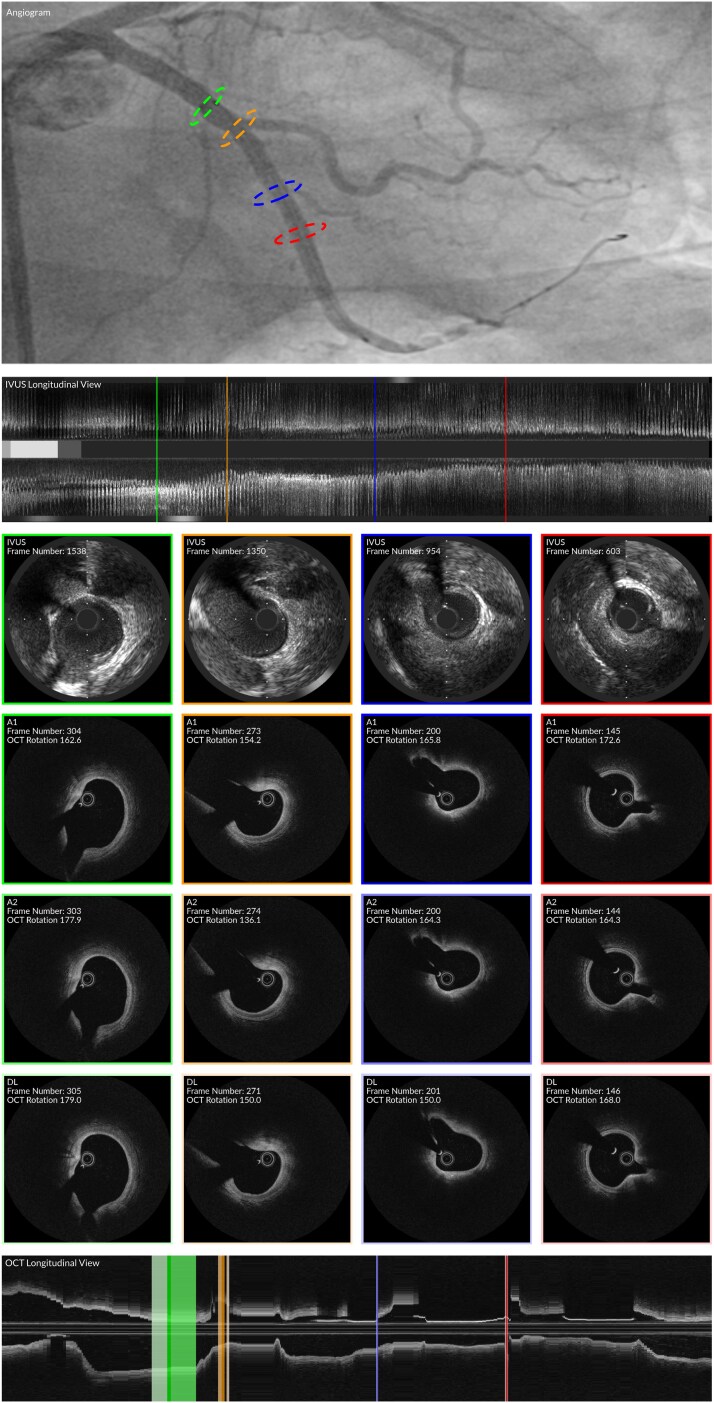
Comparison between method and expert analysts. Four frames (red, orange, blue, red) matched longitudinally and circumferentially in NIRS-IVUS and OCT by the first analyst (A1) are compared to the OCT frame and rotation predictions of the second analyst (A2) and the DL method. The asterisk (*) indicates the position of the side branch. Due to interpolation, multiple OCT frames can be matched to a single NIRS-IVUS frame. DL, deep learning.

The results from the comparison of the estimations of the expert analysts and the DL method are shown in *Table 3*, and *Figure 3*, while a case example showing the estimations of the two experts and the DL method is presented in *Figure 4*. For the longitudinal matching of NIRS-IVUS and OCT, a high correlation was found between the DL method and the estimations of the first (CCC > 0.99, *r* > 0.99) and second analyst (CCC > 0.99, *r* > 0.99). Similarly for the circumferential orientation of the OCT frames, there was a high correlation between the DL method and the two analysts (CCC > 0.90, *r* > 0.90). No statistically significant difference was found when we compared the inter-observer variability with the differences between the DL-method and the first analyst for the longitudinal matching (*P* = 0.395); however, the differences were larger when we compared the inter-observer variability and the differences between the output of the DL method to the second analyst (*P* = 0.001). In addition, there was no significant difference when we compared the inter-observer variability with the differences in the estimations of the DL and the first or second analyst for the circumferential orientation of the OCT frames (*P* = 0.244 and *P* = 0.970, respectively).

The WI for the longitudinal matching of the NIRS-IVUS and OCT frames was 0.96, and for the circumferential orientation of the NIRS-IVUS and OCT the WI was 0.97 indicating that the method has a similar efficacy with the experts in identifying correspondence between NIRS-IVUS and OCT.

The mean time needed to segment the NIRS-IVUS and OCT frames, identify matching in NIRS-IVUS and OCT and circumferentially co-register the corresponding frames was 84 s using a Nvidia 1080TiGPU with a 28-core Intel i9-7940X CPU. The time taken for the first and second analyst to identify matching and circumferentially co-register the corresponding frames was 576 and 324 s, respectively.

## Discussion

This work introduces a fully automated framework for the longitudinal and circumferential co-registration of intravascular imaging data acquired by different invasive imaging modalities with complementary strengths. Testing against the estimations of the expert analysts in a large set of NIRS-IVUS and OCT data collected in a multimodality intravascular imaging study demonstrated that the proposed method (i) is as accurate as the experts in identifying corresponding NIRS-IVUS and OCT frames, (ii) it is able to identify with a high efficacy the circumferential orientation of OCT in NIRS-IVUS, and (iii) it is fast and fully reproducible.

Accurate assessment of plaque morphology and composition is essential in stratifying cardiovascular risk and evaluating the implications of novel pharmacotherapies on atherosclerosis disease progression.^3–7,30^ Traditionally, this is performed by IVUS or OCT, two imaging modalities that have an established efficacy but also significant limitations in characterizing plaque morphology.^8,9^ IVUS is the ideal modality for quantifying the plaque burden and detecting calcific tissue but it is unable to detect the necrotic core/lipid pool tissue; NIRS can overcome this pitfall but it cannot give depth information, while OCT with its high image resolution is the best modality for measuring the cap thickness over necrotic cores/lipid pool and detecting plaque micro-characteristics associated with increased vulnerability but it has limited penetration depth and thus it does not allow visualization of the entire plaque in heavily diseased segments.^8,9^ To overcome these, multimodality imaging probes have been introduced that combine different modalities with complementary strengths and appears able to enable not only more accurate evaluation of different tissue types but also assessment of plaque biology.^10^ Combined NIRS-IVUS, IVUS-OCT, OCT-NIRS, florescence lifetime imaging-OCT, and near-infrared fluorescence-OCT systems have already been introduced in clinical practice and are expected to dominate in the future in the study of atherosclerosis. However, even these systems have limitations and are unable to provide a complete and detailed evaluation of plaque pathology. Therefore, the recent trend is to use multiple multimodality or standalone imaging probes to characterize plaque types and assess their changes over time. This strategy has allowed us to better evaluate vulnerable lesion morphology and examine the implications of emerging pharmacotherapies in plaque pathology.^11–15,30–32^

Currently, the analysis of the intravascular imaging data from these studies is performed separately for each modality as the investigators are unable to combine the collected images in a single hybrid image that will allow complete and more accurate description of plaque pathobiology. Efforts have been made over recent years to develop advanced methodologies that will be able to automatically match the intravascular imaging data acquired by different catheters.^23,33,34^ However, all of them have limitations as some require manual identification of frames with anatomical landmarks, other segmentation of the IVUS and OCT frames before matching, some others are unable to perform circumferential co-registration, while most of them have not been robustly validated against the estimations of expert analysts. The method of Molony *et al*. is the most robust approach presented in the literature for the matching of IVUS and OCT frames; however, this necessitates the segmentation of the IVUS and OCT images and appears to provide estimations that are inferior to the expert analysts.^23^

**Figure 5:**
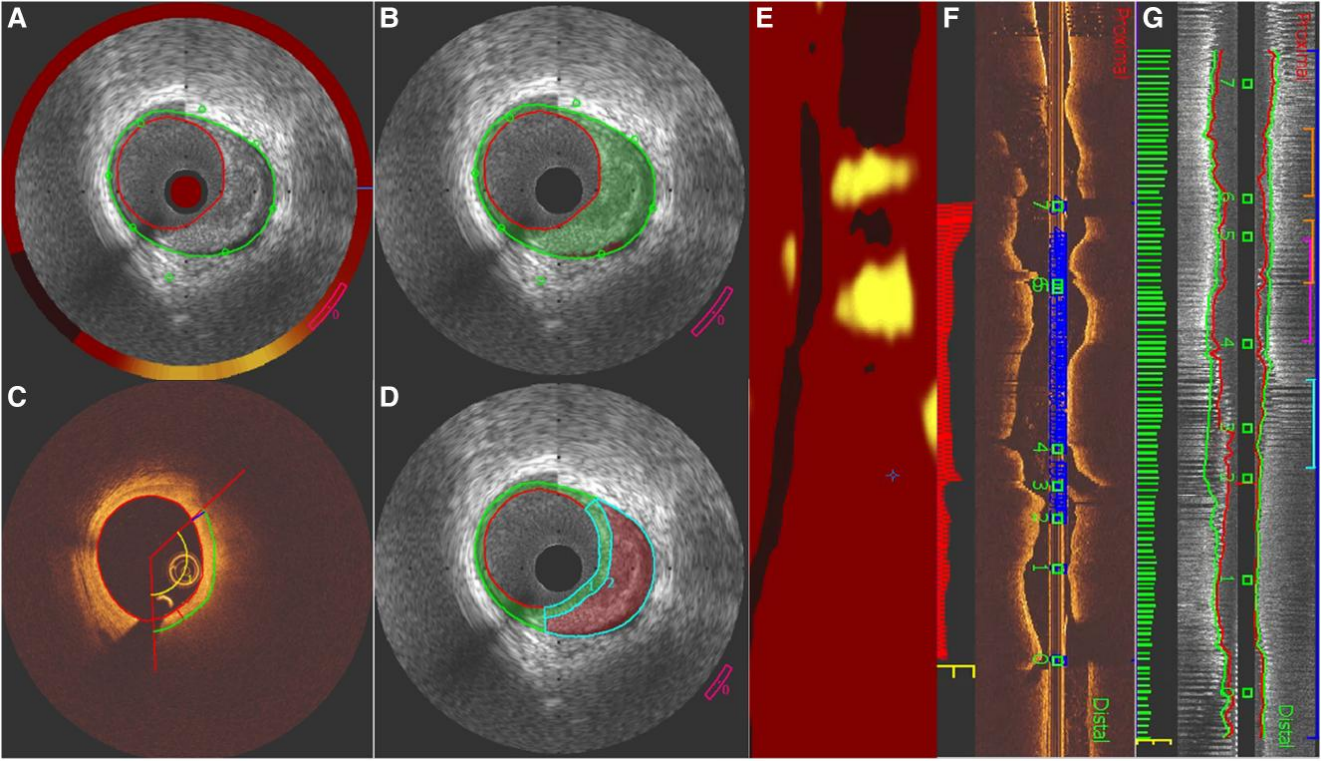
IVUS and OCT registration by expert. (*A*) NIRS-IVUS image showing lipid-rich plaque detected by near-infrared spectroscopy (NIRS), indicated by the yellow-red colour scale on the outer ring. (*B*) IVUS-derived plaque characterization using echogenicity: green represents fibrous tissue, and red represents lipid-rich plaque. (*C*) OCT cross-sectional image with manually delineated fibrous cap thickness. (*D*) IVUS image with plaque composition analysis: region 1 (blue) corresponds to the fibrous cap. (*E*) Longitudinal NIRS chemogram with yellow indicating areas of high lipid content. (*F*) Longitudinal OCT image with numbered landmarks representing side branches. (*G*) Longitudinal IVUS image showing the same landmarks as in (*F*); identical numbers indicate corresponding side branches in IVUS and OCT views.

The methodology presented in this report is the first that overcomes the above limitations, as it introduces a fully automated framework for fast and accurate matching of IVUS and OCT data. It includes effective DL solutions for the detection of the lumen borders for the identification of the origin of side branches and for the presence of calcific tissue in intravascular images and takes advantage of this information to match IVUS and OCT and estimate the rotational orientation of the OCT frames in relation to IVUS. Testing against the estimations of expert analysts in 77 vessels has shown that the proposed method is as precise as the experts in identifying corresponding frames and that it has a high performance in estimating the rotational orientation of OCT in relation to IVUS. A significant advantage of the developed method is the fact that it is fast as it is able to process and match IVUS and OCT images in only 1.5 min. This feature renders it useful in research and in particular in the analysis of large datasets collected in studies using multiple intravascular imaging systems to characterize plaque composition.^11–13,30,32,35^ The proposed approach can be combined with existing DL solutions for the detection of the external elastic lamina borders, the characterization of plaque composition in NIRS-IVUS and the estimation of the thickness of the fibrous cap in OCT and merge this information in a hybrid image that will enable more reliable quantification of different plaque components, evaluation of plaque vulnerability and of the changes in plaque phenotype after focal or systemic therapies targeting plaque evolution (*Figure 5*).^11–13,19,32,34–38^

## Limitations

Several limitations of the present analysis should be acknowledged. Firstly, the longitudinal and circumferential registration using the proposed approach is performed sequentially rather than simultaneously; therefore, temporal errors where incorrect IVUS and OCT frames are paired are likely to propagate and increase the errors in circumferential matching due to feature mismatch. In future work, we will explore landmark matching over both axes to address this issue. Secondly, the DTW approach enforces 1-to-1 longitudinal matching between IVUS and OCT sequences, this may affect the performance of the method in segments with little or no branches and calcific deposits, but matching can still occur using features from the lumen. Thirdly, the proposed method was trained and tested in data acquired by specific NIRS-IVUS and OCT systems, therefore, it is unclear whether it will be equally effective in data acquired by different catheters, and it is likely that fine tuning will be needed. In addition, the PACMAN-AMI study did not include segments with culprit lesions where often plaque rupture, erosion or an erupted calcific nodule and thrombus are present; in these challenging anatomies, it is likely our solution to be less effective. Fourthly, although the training of the DL methodologies for the detection of the lumen the side branches and the calcific tissue was performed in NIRS-IVUS and OCT datasets that had frames with common artefacts and despite the fact that the lumen segmentation method incorporated functions for the annotation of the lumen border in frames that this was not visible in its entire circumference it is likely the feature extraction to be sub-optimal in data with multiple artefacts. This may affect the co-registration of the two imaging sets; to mitigate this risk, Gaussian smoothing was applied to all features along the longitudinal axis which allowed for robust feature signal to be extracted from neighbouring frames if the feature extraction is affected by artefacts in the sampled frame. Fifthly, expert analysts must manually identify the most proximal and distal landmarks in IVUS and OCT to define the SOI. This is a relatively fast step; nevertheless, we aim to automate this in future work. Finally, we used the estimations of experts to train and test co-registration methods and not images acquired by a 3rd IVUS-OCT catheter—that would constitute the ideal reference standard—as these data were not available, and we did not examine the efficacy of the matched IVUS-OCT images that were generated by our methods in assessing plaque characteristics against histology.

## Conclusion

We introduce a fully automated framework for the longitudinal and circumferential co-registration of intravascular images acquired by IVUS- and OCT-based systems. The proposed method is fast and provides estimations that compare favourably with the expert analysts; these unique features are expected to allow its broad use in the analysis of data collected in studies of atherosclerosis that employ multimodality intravascular imaging to assess plaque phenotypes.

## Supplementary Material

ztag007_Supplementary_Data

## Data Availability

The data underlying this article will be shared on reasonable request to the corresponding author.
