# Supplementary material for: A novel framework for fully automated co-registration of intravascular ultrasound and optical coherence tomography imaging data
Source: Eur Heart J Digit Health. 2026 Jan 16;7(2):ztag007. doi: 10.1093/ehjdh/ztag007 (PMC12933311; doi:10.1093/ehjdh/ztag007)

**A novel framework for fully-automated co-registration of intravascular ultrasound and optical coherence tomography imaging data**

Xingwei He, MD^1,2,#^; Kit Mills Bransby, PhD^3,#^; Ahmet Emir Ulutas, MD^4^; Thamil Kumaran, PhD^4^; Nathan Angelo Lecaros Yap, MBChB^1^; Gonul Zeren, MD^4^; Hesong Zeng, MD, PhD^2^, Yaojun Zhang, MD, PhD^5^; Ryota Kakizaki, MD, PhD^8^; Yasushi Ueki, MD, PhD^8^; Jonas Häner, MD^8^; Antanas Gasys, MD^8^; Sylvain Losdat, PhD^9^; Andreas Baumbach, MD, PhD^1,4^; James Moon, PhD^6^; Anthony Mathur, PhD^4^; Jouke Dijkstra, PhD^7^; Qianni Zhang, PhD^3^；Lorenz Raber, MD, PhD^8,*^; Christos V Bourantas, MD, PhD^1,4,*^

^1^ Department of Cardiology, Barts Heart Centre, Barts Health NHS Trust, London, UK

^2^ Division of Cardiology, Department of Internal Medicine, Tongji Hospital, Tongji Medical College, Huazhong University of Science and Technology, Wuhan, China

^3^ School of Electronic Engineering and Computer Science, Queen Mary, University of London, London, United Kingdom

^4^ Centre for Cardiovascular Medicine and Devices, William Harvey Research Institute, Queen Mary University London, UK

^5^ Department of Cardiology, Xuzhou Third People’s Hospital, Xuzhou, China

^6^ Institute of Cardiovascular Sciences, University College London, London, UK

^7^ Division of Image Processing, Department of Radiology, Leiden University Medical Center, Leiden, The Netherlands

^8^ Department of Cardiology, Bern University Hospital, University of Bern, Bern, Switzerland

^9^CTU Bern, University of Bern, Bern, Switzerland

^#^ Equal Contributions

**Short title:**

***Address for correspondence**

Christos V Bourantas MD PhD

Consultant Cardiologist, Barts Heart Centre

Professor of Cardiology, Queen Mary University of London

Barts Heart Centre, West Smithfield, London EC1A 7BE

E-mail: c.bourantas@gmail.com

Phone: +44 20 7377 7000

Fax: +44 20 7791 9670

Lorenz Raber, MD, PhD

Professor of Interventional Cardiology, Bern University Hospital Inselspital,

Department of Cardiology, Bern University Hospital Inselspital, Freiburgstrasse 18, 3010, Bern, Switzerland

Email: lorenz.raeber@insel.ch

**Conflicts of Interests:** All authors have no conflicts of interest to declare.

**Funding:** This study is jointly funded by the British Heart Foundation; AB AM, JCM and CVB are funded by Barts NIHR Biomedical Research Centre, London, UK. XH is funded by the Opening Project of Hubei Key Laboratory of Ischemic Cardiovascular Disease (No. SZ202401).

**Supplementary Material**

*S1. Implementation of DTW algorithm for longitudinal registration and hyperparameters tuning*

DTW is performed to find the optimal temporal alignment between the NIRS-IVUS $X^{t} \in{\mathbb{\mathbb{R}}}^{n\times4}$ and OCT sequence $Y^{t} \in{\mathbb{\mathbb{R}}}^{m\times4}$. First, a distance matrix $D \in{\mathbb{\mathbb{R}}}^{n\times m}$ is computed between all pairs of frames in the sequence using a feature-weighted Euclidean distance as follows:

$$D_{ij} = \sqrt{\sum_{k=1}^{f} w_{k}^{t} \left( X_{i,k}^{t} - Y_{j,k}^{t} \right)^{2}}$$

Where $w^{t}\in{\mathbb{\mathbb{R}}}^{f}$ is a feature-wise weighting vector, and $i \in\left\{ 1,\ldots,n \right\}$, $j \in\left\{ 1,\ldots,m \right\}$ index $X^{t}$and $Y^{t}$ respectively. A DTW cost matrix $C\in{\mathbb{\mathbb{R}}}^{n\times m}$is filled where each entry represents the cumulative cost to reach that point and $C_{n,m}$is the total minimum cumulative cost of aligning the two sequences. The DTW cost matrix C is defined as:

$$C_{i,j} = D_{i,j} + \min\left\{ C_{i-1,j}, C_{i, j-1}, C_{i-1, j-1} \right\}, C_{1,1} = D_{1,1}$$

The optimal path $P$is a set of index pairs $\left( i_{p}, j_{p} \right)$of length $L$that are obtained by tracing back using the recurrence relation from $C_{n,m}$ to $C_{1,1}$:

$$P = \left\{ \left( i_{p}, j_{p} \right) \right\}_{p=1}^{L}, \left( i_{p}, j_{p} \right)= \arg\min\left\{ C_{i-1,j}, C_{i, j-1}, C_{i-1,j-1} \right\}$$

$P$defines the longitudinally match IVUS and OCT pairs that are used as input to the circumferential registration module.

We tune hyperparameters on the validation set of 65 vessels, finding optimal longitudinal feature weighting of 0.3 for lumen area, 1.5 for side branch area, 0.1 for calcium degree, and 2.5 for normalized frame position. For circumferential feature weighting, 1 for side branch, 1 for calcification angle, and 0.1 for lumen eccentricity is used.

**Supplementary Table 1:** Number of NIRS-IVUS and OCT frames included in the feature training, validation, and test set.

|  | NIRS-IVUS | | | OCT | | |
| --- | --- | --- | --- | --- | --- | --- |
|  | Training set | Validation set | Test set | Train set | Validation set | Test set |
| Lumen Segmentation | 61,665 | 6,863 | 9,099 | 62,334 | 6,484 | 8,929 |
| Side Branch Detection | 10,000 | 6,863 | 9,099 | 10,000 | 6,484 | 8,929 |
| Calcium Arc Classification | 10,000 | 6,863 | 9,099 | 10,000 | 6,484 | 8,929 |
| Co-Registration | - | 6,863 | 9,099 | - | 6,484 | 8,929 |

**Supplementary Table 2:** Training Recipes. LR=Learning Rate.

|  | Side Branch Detection | Lumen segmentation | Calcium detection |
| --- | --- | --- | --- |
| Learning Rate | 0.0001 | 0.005 | 0.005 |
| LR Scheduler | StepLR (gamma=1, stepsize=10) | Polynomial (power=0.9) | Linear |
| Batch Size | 64 | 64 | 64 |
| Epochs | 200 | 200 | 200 |
| Weight Decay | 0 | 0 | 0 |
| Optimizer | Adam | SDG | Adam |
| Loss | Cross Entropy, Smooth L1 | Cross Entropy, Dice | Cross Entropy |
| Image Resolution | 480x480 | 480x480 | 480x480 |
| Augmentation | Random Rotation (90,180,270), Brightness, Contrast | Random Rotation [-180,180], Brightness, Contrast | Random Rotation [-180,180], Brightness, Contrast |
| Confidence Threshold | 0.8 | 0.5 | 0.9 |

**Supplementary Figure 1:** Estimations of the expert analyst for the lumen, side branch and calcific tissue in paired NIRS-IVUS and OCT frames.


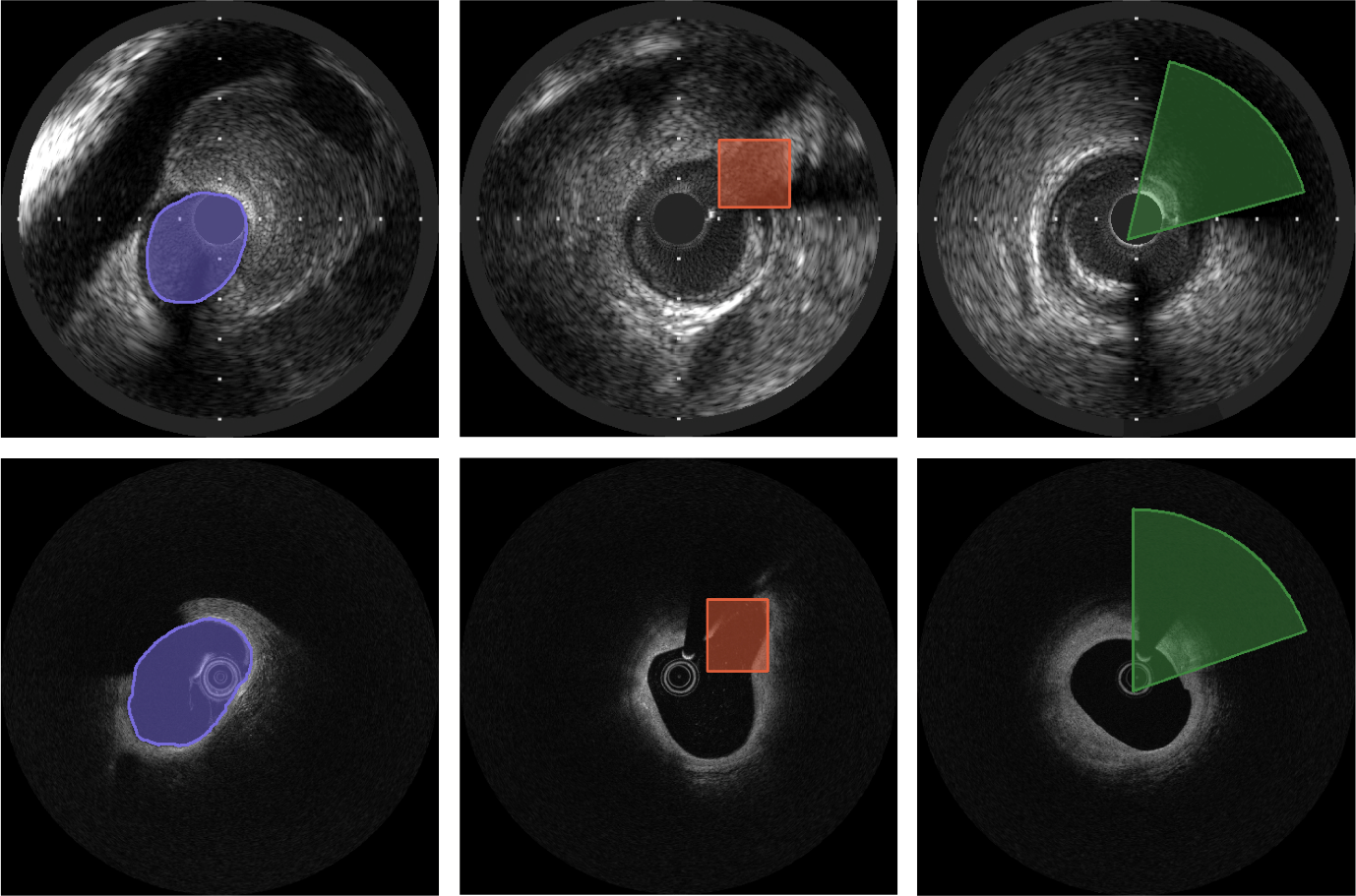


**Supplementary Figure 2:** Schematic diagram describing the architectures of each network in the feature extraction process.


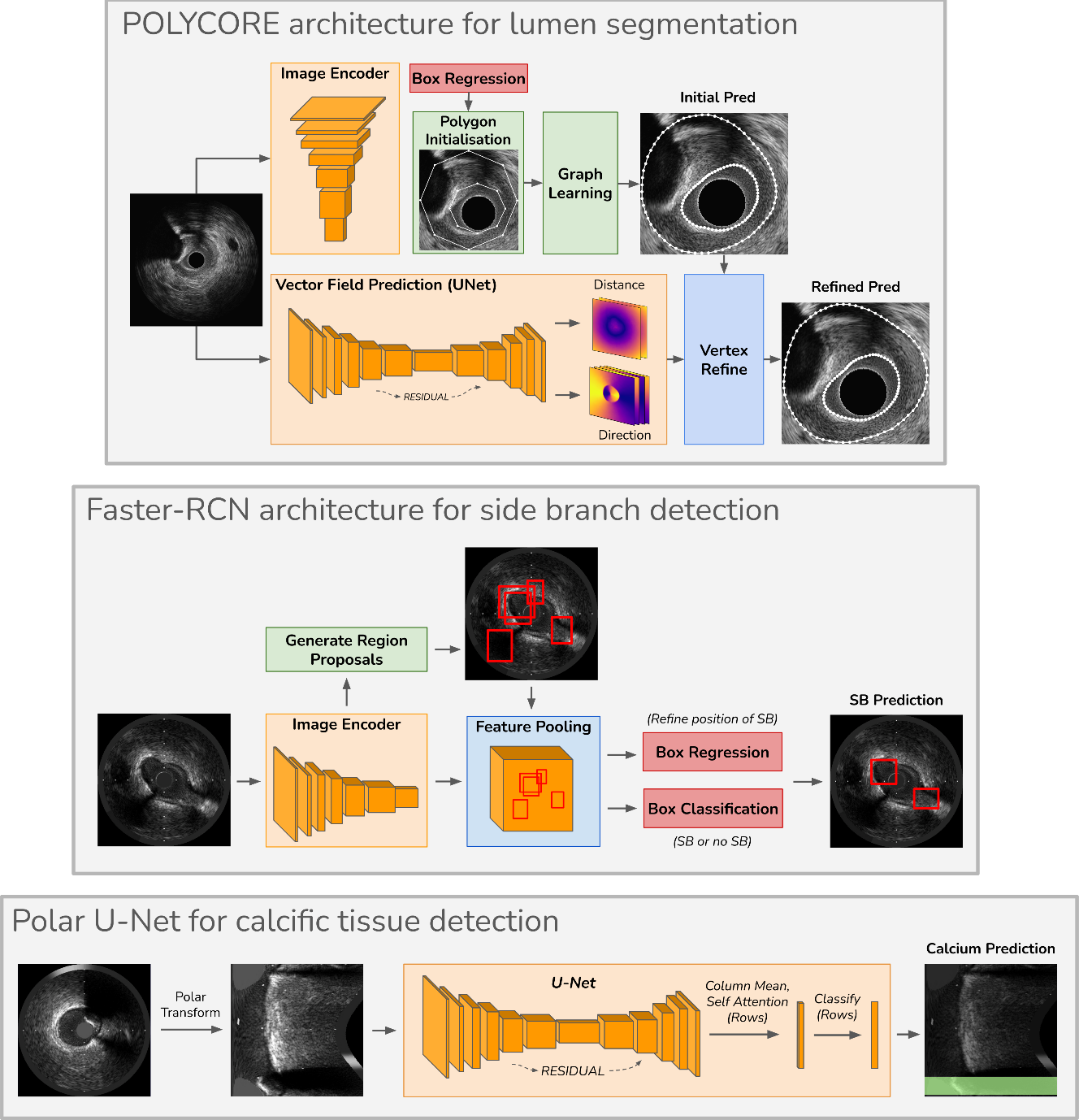


**Supplementary Figure 3:** Association between lumen areas estimated by the expert analysts and the DL methodology in NIRS-IVUS and OCT datasets. The blue line represents the regression line and the red lines the limits of agreements (±1.96 SD). The correlation coefficient (r), bias and 95% limit of agreement also presented. DL, deep learning; LoA, limits of agreement.


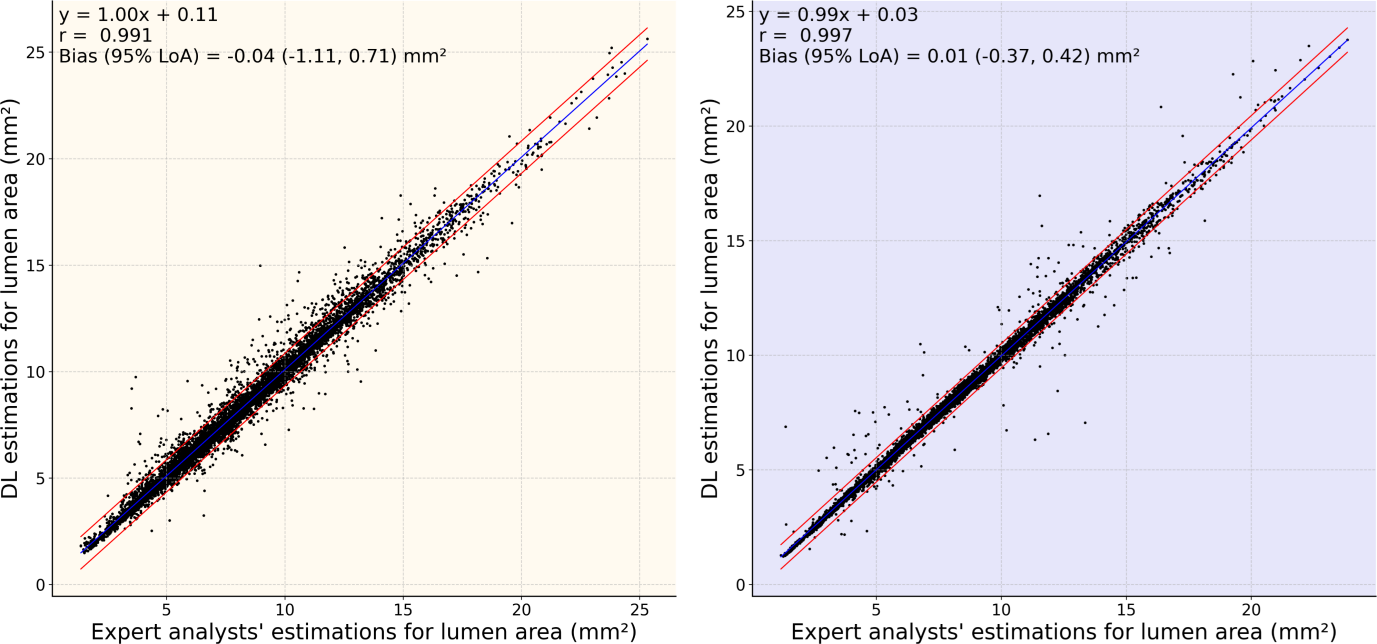

Supplement: ztag007_Supplementary_Data [file ztag007_supplementary_data.docx]
